# Comparison of Three Accelerated Oxidation Tests Applied to Red Wines with Different Chemical Composition

**DOI:** 10.3390/molecules26040815

**Published:** 2021-02-04

**Authors:** Francesca Coppola, Luigi Picariello, Martino Forino, Luigi Moio, Angelita Gambuti

**Affiliations:** Department of Agricultural Sciences, Section of Vine and Wine Sciences, University of Naples ‘Federico II’, Viale Italia, 83100 Avellino, Italy; francesca.coppola2@unina.it (F.C.); luigi.picariello@unina.it (L.P.); forino@unina.it (M.F.); luigi.moio@unina.it (L.M.)

**Keywords:** accelerated oxidation tests, wine oxidation, pigments, phenolic compounds, Aglianico, Barbera, Gaglioppo, Magliocco, Nerello

## Abstract

Background: Three accelerated oxidation tests were proposed to simulate red wine oxidation thus providing information useful to correctly manage moderate oxygen exposure of wine during aging in regard to phenolic composition and wine color. Since the results of the tests have never been compared on wines with different initial composition, the aim of this study was to find a suitable method to simulate oxidation of any still red wine. Methods: Aglianico, Barbera, Gaglioppo, Magliocco, and Nerello wines were treated with (1) three cycles of air saturation, (2) the addition of hydrogen peroxide, and (3) the addition of acetaldehyde. Changes in chromatic characteristics and phenolic composition were determined by spectrophotometric and HPLC methods. Results: Important differences in the behavior of the different wines were detected: the highest formation of polymeric pigments was observed in Barbera and Aglianico wines. In contrast, Gaglioppo and Magliocco wines showed a lower variability before and after the oxidation probably due to the lower anthocyanin/tannin ratio. Among the accelerated oxidation tests applied, no significant differences in color parameters and phenolic composition were detected in samples treated with the addition of H_2_O_2_ and the air saturation method. Conclusion: The study demonstrated that H_2_O_2_ addition is a successful tool to predict the evolution of different phenolic compounds during the air saturation treatment of wines.

## 1. Introduction

Red wine quality is strongly influenced not only by the characteristics of the starting raw materials, but also by the transformation and ageing techniques. In each step of the transformation phase, wine components undergo changes and interactions in which oxygen plays an essential role. Starting from alcoholic fermentation, oxygen is consumed, first by yeasts that produce sterols to acquire greater resistance to alcohol and optimize their ability to absorb nitrogen compounds [1], then during the storage and ageing process, mainly by phenolic compounds that determine important changes in the sensory profile of wines, in particular in terms of color, bitterness, and astringency. On the other hand, exposures to excessive amounts of oxygen give red wines unwanted aromatic profiles, such as oxidative off-flavors and colors including orange hues [2].

For these reasons, the management of the exposure to oxygen, critical for each phase of the winemaking and ageing process of wines, represents an important issue for winemakers. In fact, the reactions in which oxygen is involved characterize the aging of wines in every kind of container, including steel containers, barrels, and bottles, and affect their quality [3]. Moreover, given the variability of the chemical composition of different wines, it is very difficult to accurately predict the fate of a product that is exposed to controlled and, even worse, uncontrolled quantities of oxygen.

The scientific literature is rich in papers dealing with the reactions that occur when wine is exposed to oxygen. Once oxygen diffuses in wine, it oxidizes Fe(II) to generate Fe(III) and hydrogen peroxide which is further catalyzed by the presence of copper. Subsequently, Fe(III) oxidizes ortho-diphenol units, typical of some wine phenolic compounds, to quinones. Hydrogen peroxide (H_2_O_2_) together with Fe(II) and/or Cu(I) starts the Fenton reaction.

A critical product of this latter reaction is the hydroxyl radical (HO•) a powerful oxidant capable of oxidizing indiscriminately many organic compounds. The main product of the Fenton reaction is therefore acetaldehyde deriving from the oxidation of ethanol. In this chemical context, sulfur dioxide plays a pivotal role, as it removes hydrogen peroxide, thus preventing the Fenton reaction from occurring. Furthermore, SO_2_ reduces quinones back to ortho-diphenols (catechol) and so it prevents quinones from reacting further [2]. Finally, it is reversibly bound (1:1) to acetaldehyde. 

The oxidative cascade in red wine is therefore characterized by three highly reactive compounds: oxygen, hydrogen peroxide, and acetaldehyde, but the phenolic composition of red wines needs to be taken into account. Phenolics in wines vary in terms of quality and quantity of either low or high molecular weight molecules and they on one hand act as natural antioxidant by quenching free radicals, but on the other, they reduce Fe(III) back to Fe(II), thus causing the oxidation to proceed. It follows that it is of critical importance to evaluate how different typologies of red wines react to oxidation.

In literature, there are numerous studies on the oxidative evolution of red wines by means of accelerated aging assessment tests that are very different from each other. Some are based on exposure to high temperatures, other on enzymatic tests, the addition of hydrogen peroxide [4,5], and the addition of exogenous acetaldehyde [6,7]. Among them, one of the most employed is the evaluation of oxygen consumption after successive saturation to investigate the behavior of wine pigments, tannins, and aroma compounds [8,9]. To the best of our knowledge, only recently a comparison among three of these tests has been made on Shiraz wine [5] and never have different red wines been considered.

In this paper, we report on the effect of three different systems of oxygen addition to wine with the purpose of developing a tool to predict, in a short time, the evolution of phenolic compounds due to moderate oxidation in wines. The effect of direct oxygen consumption after three cycles of saturation (sat) and hydrogen peroxide (H_2_O_2_) and acetaldehyde addition (AtCH) was evaluated in five red wines (Aglianico, Barbera, Magliocco, Gaglioppo, and Nerello) characterized by very different initial phenolic compositions in terms of monomeric and polymeric pigments as well as uncolored phenolics.

## 2. Results and Discussion

Wine oxidations are related to a complex series of chemical modifications, mainly involving phenolic components. In this work, we evaluated the effect of oxidative events in five red wine samples, which were selected as to cover a wide range of wine types characterized by different phenol compositions. The primary purpose of the research was to assess the response of wine to oxidative injury based on the distribution of wine phenolics. To this aim, we simulated the oxidative evolution by resorting to air saturation as well as to the addition of acetaldehyde or H_2_O_2_.

**Figure 1:**
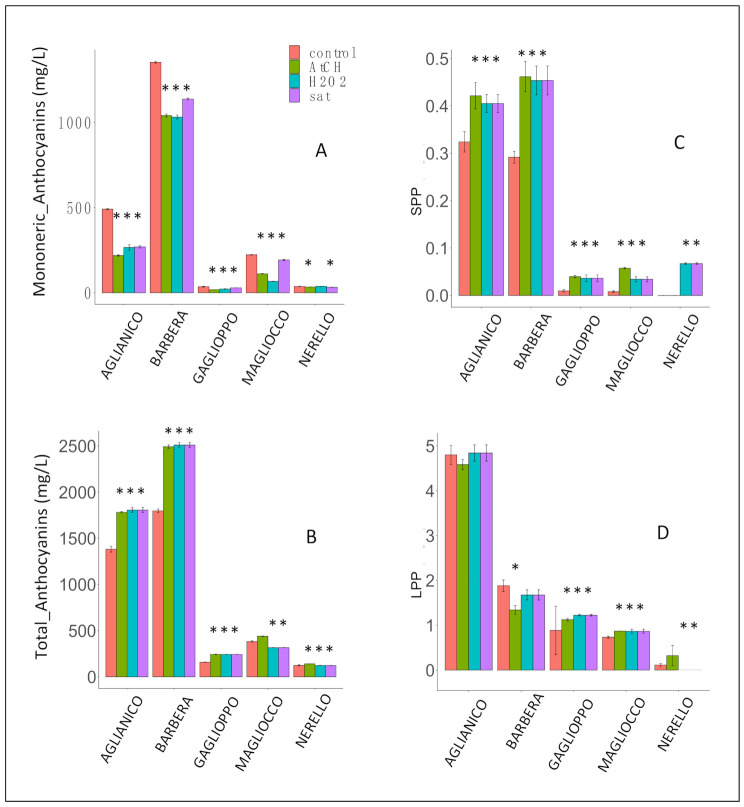
Variation of pigments in response to oxidative tests in different wines (Aglianico; Barbera; Gaglioppo; Magliocco; Nerello; Mascalese). (**A**) Sum of monomeric anthocyanins; (**B**) total anthocyanins; (**C**) polymeric pigments with a low degree of polymerization; (**D**) polymeric pigments with a high degree of polymerization. Asterisks (*) indicate significant differences between treated wine and its control (*p*-value less than 0.05).

The response of the wine phenolic compounds to oxidation was determined by evaluating the variation of anthocyanins, polymeric pigments, chromatic features, the protein reactive tannins, and vanillin reactive flavans. In regard to the sulphite-driven protection of red wines assayed, we used samples poorly protected as it often occurs in wineries after the centrifugation and filtration treatments and before the bottling. On the basis of the different values of sulfur dioxide in wines (Table 1), we could refer to all wines as poorly protected by SO_2_ because the official method of analysis used to determine the free SO_2_ in red wines systematically overestimates both the molecular and free SO_2_ in red wines due to the presence of anthocyanins [10]. However, it is worth emphasizing that the complex between SO_2_ and anthocyanins readily dissociates thus making such initially bound SO_2_ available during oxidation. This is especially true for Barbera that is the richest in anthocyanins (Figure 1A).

### 2.1. Variation of Pigments and Chromatic Characteristics

As expected, oxidation events induced drastic chromatic variations in wines due to changes in their pigment compositions (Figure 1 and Table 2). The total content of monomeric anthocyanins, determined by HPLC-based analyses, significantly decreased in all the wine samples compared to their respective controls (Figure 1A and Table A1). Slight differences among the oxidative treatments were detected. In contrast, oxidative treatments induced an increase in Aglianico and Barbera wines of the total anthocyanins, determined by the Harbertson–Adams assay [11]. This was likely the consequence of the formation of new pigments exhibiting absorption bands close to λmax of anthocyanins (520 nm) (Figure 1B) [12]. The amount of the newly formed pigments overcame the decrement of native anthocyanins. Total pigments increased at comparable levels due to oxidative treatments and appeared to be independent of the polyphenolic composition of wines. In the Aglianico and Barbera wines, the increased concentration of total pigments was more evident than for the other wines because of the relatively higher number of native anthocyanins. In fact, the most important anthocyanin derivatives are new polymeric pigments generated either by direct condensation reaction between anthocyanins and flavanols or by the ethanal action forming flavanol-ethyl-anthocyanin adducts [12]. In this study, polymeric pigments were determined as two different classes, namely, large polymeric pigments (LPP) and small polymeric pigments (SPP), separately determined by the Harbertson–Adams assay [11]. SPP increased in all the treated wine samples apart from Nerello_ATCH._ (Figure 1C). Given that Nerello turned out to be quite poor in both flavanols and anthocyanins, the formation of ethylidene-bridged pigments was not significant, while in Nerello_H_2_O_2__ and Nerello_sat_ the oxidative environment may have favored the direct condensation between anthocyanins and flavanols. Additionally, except Barbera_ATCH_, the level of LPP turned out to be practically unaffected by oxidative tests (Figure 1D). The evidence that newly formed pigments were small-sized condensation products, which are not precipitated by BSA, would suggest that kinetically the formation of small tannins would be favored over the time the experiments were carried out. The decrease of LPP in Barbera_ATCH_ could be due to the fact that molecules with higher polymerization degrees are not detected by the BSA-based assay, even though their formation cannot be ruled out altogether.

The CIEL*a*b* colorimetric model defines a panel of descriptors (coordinates) for the chromatic variation of wines. The values of the different color coordinates measured for the oxidative treatments are shown in Table 2.

Lightness (L*) decreased for oxidized wines due to a progressive darkening. The a* coordinate (green-red color component) increased for all wines, pointing out that wines acquired more intense red shades, likely related to the neoformation of red pigments [12]. The b* coordinate (blue-yellow color components) increased in Aglianico, Barbera, and Nerello giving more orange-yellow pigments while it decreased in Gaglioppo and Magliocco, suggesting that, for these wines, there was no appreciable formation of orange-yellow pigments resulting from oxidation of flavanols or from anthocyanin degradation [13]. Chromacity (C) increased after oxidation for all wines. A similar trend was even detected by Avizcuri et al. [14] after aging in bottle of sixteen commercial red wines. During the oxidative tests, it is possible that a change in color occurs due to the removal of sulfur dioxide from the colorless derivatives previously formed by sulfite and anthocyanins [15]. Furthermore, the formation of new pigments could be responsible for the observed effects [12]. ΔE provides a useful indicator of the effects of oxidation on human visual perception [16]. In our case, this parameter confirmed that oxidation tests produced noticeable (Nerello wine) or remarkable (Aglianico, Barbera, Gaglioppo, and Magliocco wines) color variations. Data on color intensity and hue confirmed the trend detected for CIELab coordinates (Figure A2). The color alteration was particularly high for Barbera wine (ΔE = 16.49–18.56).

### 2.2. Variation of Protein Reactive Tannins and Vanillin Reactive Flavans

**Figure 2:**
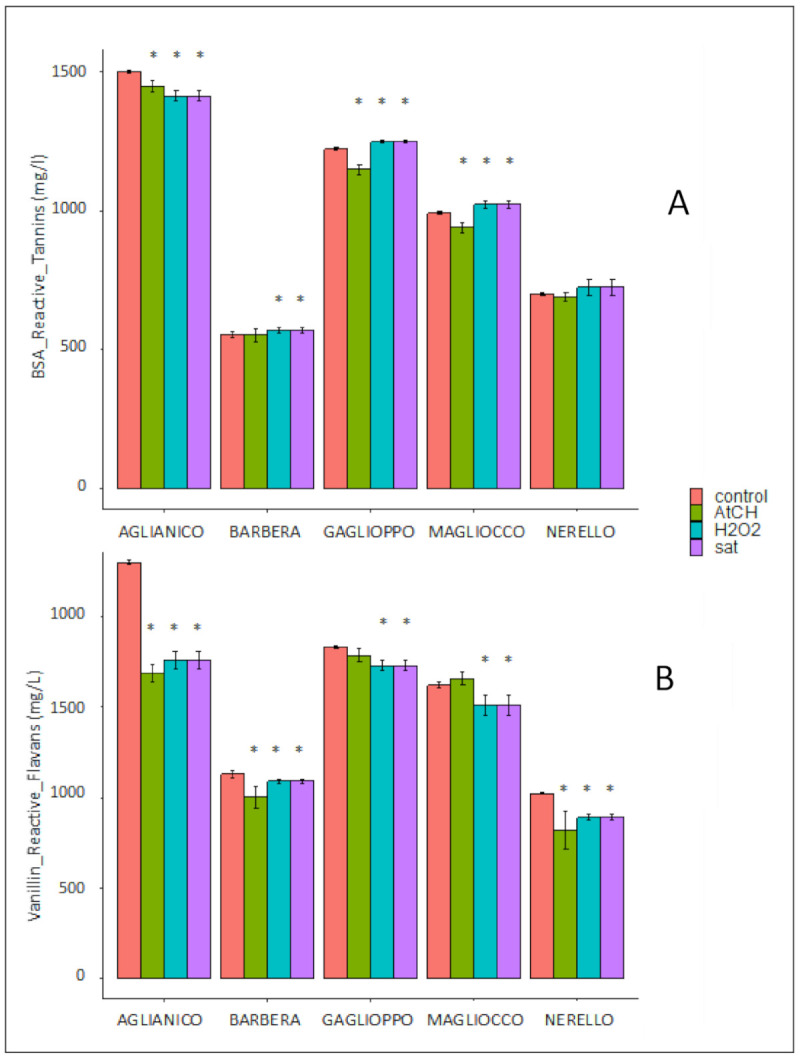
Tannins content in treated wines (Aglianico; Barbera; Gaglioppo; Magliocco; Nerello; Mascalese); (**A**) BSA reactive tannins (mg/L), (**B**) vanillin reactive flavans (mg/L) content in wines before and after treatments. Asterisks (*) indicate significant differences between treated wine and its control (*p*-value less than 0.05).

Protein (BSA) reactive tannins, determined by the Harbertson–Adams assay [11], were scarcely affected by oxidative treatments in all wines (Figure 2A). This finding suggests that accelerated oxidation is not able to induce condensation between flavan-3-ols to generate new oligomeric tannins. Following the oxidation tests, we observed that mainly short polymeric pigments were affected. In our opinion, the above results confirmed that anthocyanins are indeed the compounds crucially involved in modulating the response of red wines to moderate oxidation [8]. Slight differences between control wines and treated ones were observed. Wine A was the only wine where a small decrease of tannins was detected after oxidation. This was likely due to its peculiar A/T ratio as already observed in a previous study [8]. For Gaglioppo and Magliocco, only the acetaldehyde addition determined a decrease of the BSA reactive tannins. In order to understand the effect of the different treatments on the acetaldehyde content of wines, this molecule was determined by HPLC analysis after derivatization of wine samples with DNPH. No clear trend either upward or downward was shown (Figure A3) probably owing to the great reactivity of this electrophile compound in such complex media which are red wines. It should also be taken into account that acetaldehyde instantly reacts with all the free SO_2_ present in wine [17]. Further studies in either model solutions or real wines could help the understanding of this important issue. Vanillin reactive flavans (VRF) give an indirect indication about the variation of the polymerization degree of condensed tannins [18]. Vanillin is reactive towards the C6 and C8 positions of free flavan-3-ols, whereas it is less correlated with long polymeric flavan-3-ols (mean polymerization degree mPD > 4), which have the C6 and C8 carbon positions already involved in inter-monomers covalent bonds. We observed a reduction of VRF in all the examined wines; this was particularly remarkable for the Aglianico wine probably due to a lower pH value compared to the other wines. In fact, it is well known that a low pH favors any carbonyl functionality reactivity, including acetaldehyde, with nucleophilic positions of wine phenolics (Figure 2B).

The reduction of VRF, the increased amount of SPP, the slight variation in BSA reactive tannins observed in the oxidized wines confirmed the hypothesis that condensation between anthocyanins and flavan-3-ols together with the slight increase of degree of polymerization would be occurring, rather than an ex novo polymerization of catechins.

### 2.3. Application of Accelerated Oxidation Tests

**Figure 3:**
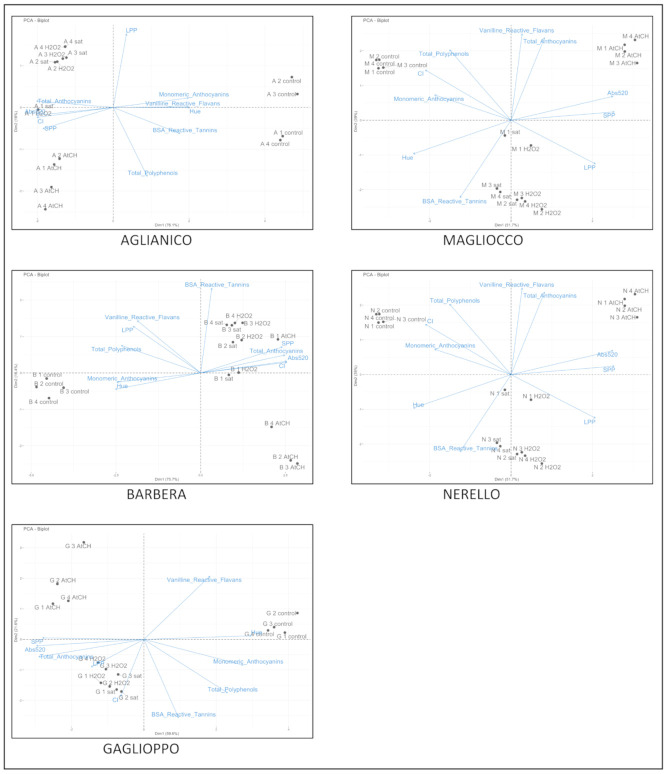
PCA score biplot of the first two PCs of data set of all phenolic compounds and chromatic characteristics in control and oxidized samples for each monovarietal wine (Aglianico, Barbera, Gaglioppo, Magliocco, Nerello).

A principle component analysis was performed to assess the effects of oxidative treatments on phenolic composition and chromatic characteristics of each monovarietal wine considered (Figure 3). For all wines, SPP and Abs 520 nm are the common drivers for PC1. For each kind of red wine, different drivers are associated to PC2, for Aglianico they are LPP and total polyphenols, BSA reactive tannins for Barbera, monomeric anthocyanins for Gaglioppo, VRF and total anthocyanins for Magliocco and Nerello.

For Aglianico, Barbera, and Gaglioppo, the control wine was always well separated from the treated ones along the first component. The PCA showed that, for these wines, values associated with oxidized wines are more closely associated with SPP and total anthocyanins. During oxidation of red wine, the formation of more stable complex pigments occurs [12,19], the good separation along the first component (from 59.6% to 76.1% of total variance explained) is therefore easily justified. For Aglianico wines, those treated with sat and H_2_O_2_ treated are well grouped and are associated with LPP. This result is not surprising because a shift towards more polymerized structures during oxidation is expected [20].

The analysis of PCA allowed also to discriminate accelerated oxidation tests from control wines in the case of Magliocco and Nerello wines but the separation was not so high as for Aglianico, Barbera where the 90.7% of total variance was explained. In contrast, AtCH tests differ from other tests and are associated with Abs at 520 nm. This is not surprising because acetaldehyde is highly reactive towards monomeric anthocyanins and the high correlation between AtCH tests and Abs at 520 nm confirms previous studies in which an increase in color intensity in red wines due to the effect of acetaldehyde was observed in model solutions [19,21] and red wine [6]. The chemical reasons for this behavior are linked to the fact that the main products of the reaction between acetaldehyde and anthocyanins are ethyl-linked anthocyanin-flavanols adducts (A-e-F) and vitisin B [12]. Several authors have in fact found that the formation of ethylene linked Mv3gl-catechin dimers caused a shift towards blue tint of solution containing anthocyanins [22]. Nonetheless, it should also be considered that these reactions are not the only occurring during oxidative processes linked to wine aging. Recently, in a study in which anthocyanins and their derivatives of 234 bottled commercial wines from 1 to 23 years aged were analyzed, a great variation in the evolution pattern among anthocyanin derivative classes was detected [23]. In particular, vitisin B and A-e-F products degraded in almost five years. Moreover, Escribano-Bailón et al. [24] showed that A-e-F are more likely to degrade in aqueous solution than anthocyanins due to the facile cleavage of the ethyl bridge. In addition, previous studies have shown that SO_2_ including that deriving from the acetataldehyde-bisulfite adducts is eventually consumed through oxidation reactions [17], hence an increase of acetaldehyde in solution must be also considered. On the basis of what was reported above and of our results, we can conclude that the use of AtCH addition to predict wine oxidation is less accurate than the addition of H_2_O_2_ for a correct estimation of pigments evolution and, in general, of wine evolution under moderate oxidation. Therefore, given the consistence of the response to H_2_O_2_ oxidation test with those based on air saturation, we propose the employment of the H_2_O_2_ test to predict the phenolic and color evolution of red wines under moderate oxidation since it is quite easy and fast to perform compared to air saturations and handling of acetaldehyde. Hence, it could be regarded as a preferential tool to assess wine response to oxidations.

**Figure 4:**
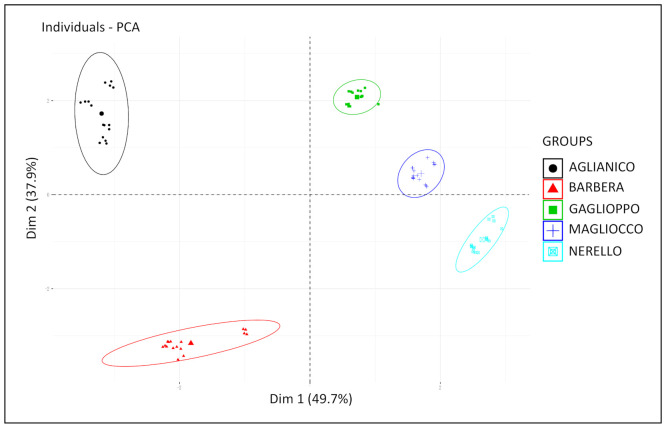
PCA score plot of the first two PCs of whole phenolics and chromatic characteristics data set.

The application of accelerated oxidation tests to the five monovarietal wines considered was instrumental to understand as to whether the oxidation could determine changes in the phenolic composition of red wines to such an extent to limit the possibility of distinguishing the wines from each other. A principal component analysis was then performed and, as it can be seen from the PCA score plot (Figure 4), wine samples tended to clusterize regardless of the oxidation treatment applied.

Anthocyanin-poor wines (Gaglioppo, Magliocco, and Nerello) turned out to be clustered on the same side of the plot and they were differentiated by the variables VRF, BSA reactive tannins, total polyphenols, and acetaldehyde. In turn, Barbera and Aglianico occurred on opposite sides with respect to dimension 2 (characterized by parameters such as SPP, total anthocyanins, monomeric anthocyanins, CI, and Abs 520). It is interesting to observe that Gaglioppo and Magliocco wines showed a lower variability before and after the oxidative tests while Barbera, Aglianico, and, to a lesser extent, Nerello changed after the oxidation. This could indicate a reactivity of Barbera and Aglianico towards oxygen that Gaglioppo and Magliocco do not have anymore. As when red wines age, they possess lower quantities of reactive phenolic compounds [23], it is possible to adopt these accelerated oxidation tests to predict the “possible evolution of color and pigments” of a red wine. In particular, the changes in hue, total anthocyanins, and VRF should be considered. The more a red wine treated with H_2_O_2_ changes these parameters increasing total anthocyanins and preserving a hue below 1 (Abs520 nm > Abs420 nm), the more the moderate exposure to oxygen is not detrimental for this wine. A concomitant significant decrease of VRF could be also associated to a possible variation in the structure of polymeric flavonoids affecting mouthfeel. Thus, the enologists could adopt aging strategies (micro-oxygenation, closures with specific oxygen permeating capacity, and SO_2_ protection) more adapted to the phenolic composition of this red wine.

In conclusion, oxygen saturation cycles and hydrogen peroxide addition tests have the same impacts on most of the wine chromatic properties and phenolic composition of the treated wines. Thus, both can be used as suitable methods to simulate red wine oxidation and to allow a discrimination among wines based on their possible evolution under oxygen exposure. 

## 3. Materials and Methods

### 3.1. Wines

Five red wines of southern Italy made from different indigenous grape varieties—Aglianico, Barbera, Gaglioppo, Magliocco, and Nerello—were used in the study. Monovarietal wines were produced with Nerello Mascalese and Magliocco grapes produced in the Calabria region (Crotone, Italy) by Marrelli Wines Cantina e Vigneti (Crotone, Italy) with a standard industrial process during 2019 vintage. The blend Gaglioppo-Magliocco (50%−50%) was produced in the same area in 2018 vintage. Aglianico wine was produced in 2019 vintage by Masseria Della Porta, and Barbera wine was produced in 2019 by A’cancellera winery (BN, Italy). Table 1 shows the basic parameters of wines before treatments, after centrifugation and 0.45 mm filtration. Microbiological plating showed that wines were not containing yeasts and bacteria after filtration. As the aim of this study was to simulate the possible application of these tests in a winery in the pre-bottling phase, values of free SO_2_ were in the range 10 ± 8 mg/L as it often occurs in this phase. Wine parameters were determined by OIV methods of analysis [25].

### 3.2. Accelerated Oxidation Tests

**Figure A1:**
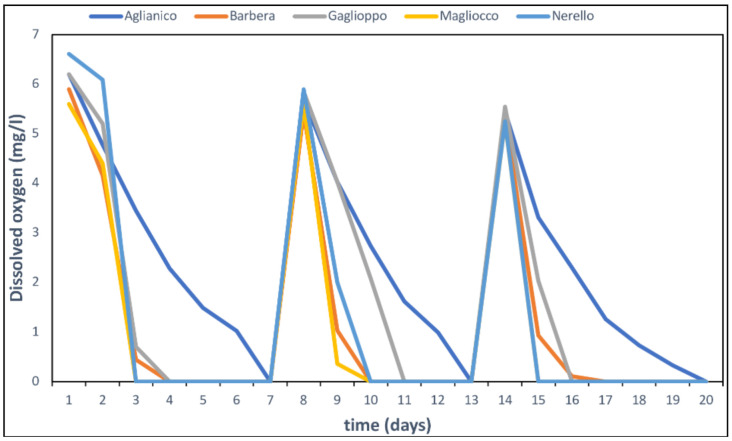
Dissolved oxygen in wines treated with three cycles of saturation (sat).

**Figure A2:**
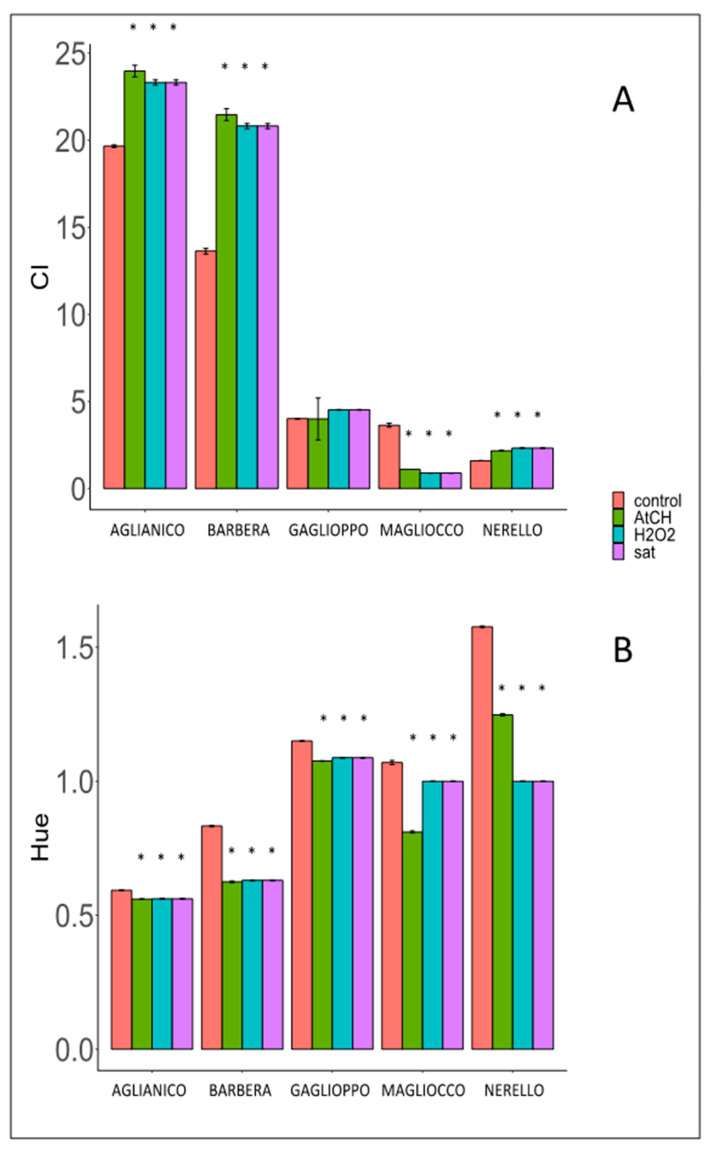
(**A**) Color intensity (CI) and (**B**) hue of wines before and after oxidative treatments. Asterisks (*) indicate significant differences between treated wine and its control (*p*-value less than 0.05).

**Figure A3:**
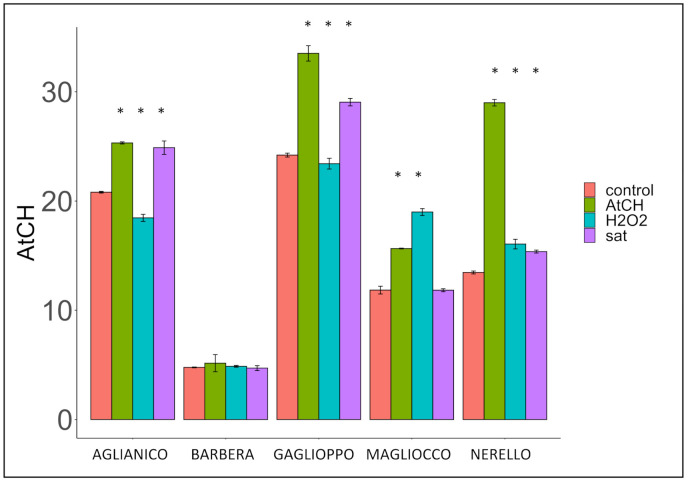
Acetaldehyde content in wines before and after oxidative treatments. Asterisks (*) indicate significant differences between treated wine and its control (*p*-value less than 0.05).

Three accelerated oxidation tests were compared: oxygen saturation (sat), hydrogen peroxide addition (H_2_O_2_), and acetaldehyde addition (AtCH). Saturation test (sat): The oxidation test consisted of three consecutive air saturation cycles. The chemical composition of wines before and after the oxidation was extensively characterized. The procedure used was the same of Gambuti et al. [8]. Briefly: Two 1 L bottles of each wine containing PSt3 oxygen sensors (Nomacorc SA, Thimister-Clermont, Belgium) were saturated by adding a gentle flow of air through a mini-compressor for 15 min until the oxygen level of the wine reached 6.6 mg/L. Bottles were previously autoclaved. Wines were stored in an incubator in the dark at 25 °C, and the dissolved oxygen level was monitored at least once a day with a Nomasense oxygen analyzer from Nomacorc S.A. (Thimister-Clermont, Belgium). The oxidation cycle was considered finished once O_2_ levels dropped to 10% of the initial concentration (Figure A1). Hydrogen peroxide test (H_2_O_2_): Treated wines were obtained by adding 19 mg/L of H_2_O_2_ (eq. to 18 mg/L O_2_) (30% Fluka, Sigma Aldrich Chemie GmbH Steinheim, France) at 20 °C, considering a 1:1 stoichiometry of oxygen to hydrogen peroxide. Acetaldehyde test (AtCH): Treated wines were obtained by adding 24.7 mg/L (eq. to 18 mg/L O_2_) of acetaldehyde (ACS reagent ≥99.5%. Sigma Aldrich Chemie GmbH, Steinheim, France) at 20 °C. Furthermore, in this case, a 1:1 stoichiometry of oxygen to acetaldehyde generation was considered. H_2_O_2_ and AtCH were performed by transferring the red wines in autoclaved pyrex tubes previously saturated with nitrogen. Just after filling the tubes with wine, they were closed with a cork by using sealing wax. When AtCH and H_2_O_2_ tests were carried out, the solutions containing the two reagents (respectively acetaldehyde and hydrogen peroxide) were added to the wines through the corks by using a sealed hypodermic syringe attached to them.

All the tests were performed to simulate a further contact of wine with 18 mg/L of oxygen and assuming that the consequent oxidation process produced exclusively hydrogen peroxide or acetaldehyde.

Analyses of treated wines were carried out 15 days after the tests were performed. All tests were made in duplicate.

### 3.3. Spectrophotometric Analyses

Color intensity (Abs 420 nm + Abs 520 nm + Abs 620 nm) and hue (Abs 420 nm/Abs 520 nm) were determined spectrophotometrically using a UV spectrophotometer. All analyses were carried out in duplicate. The CIELAB parameters (L*, a*, b*) were determined by using the software Panorama (PANORAMA SOFTWARE UPGRADE PATH), following the recommendations of the Commission Internationale de L’Eclariage (CIE). Color differences (ΔE⁄ab) were calculated as the Euclidean distance between two points in the 3D space defined by L*, a*, and b*. Total anthocyanins, bovine serum albumin (Sigma Aldrich SRL, Milano, Italy), reactive tannins (BSA reactive tannins), short polymeric pigments (SPP), and large polymeric pigments (LPP) were determined by the Harbertson–Adams assay [11]. Briefly, pH changes permitted the evaluation of total anthocyanins, and small polymeric pigments (SPP) and large polymeric pigments (LPP) were obtained by combining analysis of supernatant obtained after protein precipitation using bovine serum albumin BSA (Spectrum Chemical, Gardena, CA, USA) with the bisulfite bleaching of pigments. Vanillin reactive flavans (VRF) were determined as described by Gambuti et al. [8]. Briefly, 125 μL of wine previously diluted 1 to 10 with methanol were added with 750 μL of a solution of vanillin (4% in methanol). After 5 min, 375 μL of concentrated hydrochloric acid was added at 4 °C. After a 15-min incubation of the mixture at room temperature (20 °C), the absorbance was determined at 500 nm against a blank in which pure methanol was used instead of the solution of vanillin.

All experiments were carried out in duplicate and two analytical replicas were performed.

### 3.4. High-Performance Liquid Chromatography Determination of Acetaldehyde

Acetaldehyde was determined by HPLC after derivatization reaction with 2.4-dinitrophenylhydrazine reagent (Aldrich chemistry) according to the OIV method of analysis [25]. Wine sample aliquots (100 μL) were dispensed to a vial, followed by the addition of 20 μL of freshly prepared 1120 mg/L SO_2_ solution, 20 μL of 25% sulfuric acid (Carlo Erba reagent 96%), and 140 μL of 2 g/L 2.4-dinitrophenylhydrazine reagent. After mixing, the solution was left to react for 15 min at 65 °C and then promptly cooled to room temperature. Carbonyl hydrazones were analyzed by HPLC using a HPLC Shimadzu LC10 ADVP apparatus (Shimadzu Italy, Milan), consisting of a SCL-10AVP system controller, two LC-10ADVP pumps, a SPD-M 10 AVP detector, and an injection system full Rheodyne model 7725 (Rheodyne. Cotati. CA) equipped with a 50 µL loop. The separation was carried out on a Waters Spherisorb column (250 × 4.6 mm, 4μm particles diameter) equipped with a guard column. Optimum efficiency of separation was obtained using a flow rate of 0.75 mL/min, and the column temperature was of 35 °C. Mobile phase solvents were (A) 0.5% formic acid (Sigma Aldrich ≥ 95%) in water milli-Q (Sigma Aldrich) and (B) acetonitrile (Sigma Aldrich ≥ 99.9%); gradient elution protocol was 35% B to 60% B (t = 8 min), 60% B to 90% B (t = 13 min), 90% B to 95% B (t = 15 min. 2-min hold), 95% B to 35% B (t = 17 min, 4-min hold), total run time, 21 min. Eluted peaks were compared with derivatized acetaldehyde standard. All experiments were carried out in duplicate and two analytical replicas were performed.

### 3.5. High-Performance Liquid Chromatography Analyses of Anthocyanins

The separation of the monomeric anthocyanins was performed according to the OIV method of analysis [25] in the HPLC system previously described by using a column heating device set at 40 °C, with a C18 column, Waters Spherisorb column (250 × 4.6 mm, 4 μm particles diameter) with pre-column. All the samples were filtered through 0.45 μm filters (Durapore membrane filters, Millipore—Ireland) into glass vials and immediately injected into the HPLC system. A 50 μL loop was used. Elution was carried out by using a flow rate of 0.80 mL/min. Eluents were solvent A consisting of water milli-Q (Sigma Aldrich)/formic acid (Sigma Aldrich ≥ 95%)/acetonitrile (Sigma Aldrich ≥ 99.9%) (87:10:3) *v/v/v*, and solvent B consisting of water/formic acid/acetonitrile (40:10:50) *v/v/v*. The following gradient was used: zero-time conditions 94% A and 6% B. after 15 min the pumps were adjusted to 70% A and 30% B, at 30 min to 50% A and 50% B, at 35 min to 40% A and 60% B, at 41 min end of analysis, to 94% A and 6% B. For calibration, the external standard method was used: the calibration curve was plotted for the malvidin-3-monoglucoside (Extrasynthese, Lyon, France) on the basis of peak area. The concentration of the following monomeric anthocyanins was determined: delphinidin 3-glucoside, cyanidin 3-glucoside, peonidin 3-monoglucoside, malvidin 3-glucoside, malvidin 3-(6^II^-acetyl)-glucoside, malvidin 3-(6^II^-coumaroyl)-glucoside. The concentration was expressed as mg/L of malvidin-3-monoglucoside. All experiments were carried out in duplicate and two analytical replicas were performed.

### 3.6. Statistical Analysis

Data were expressed as the average and standard deviation of measurements performed for the different wines and treatments. The influence of treatments was analyzed by a non-parametric procedure by aligned ranks transformation ANOVA to determine whether there was an overall significant difference in group means. Post hoc pairwise comparisons of levels within single factors were conducted among all levels within each factor. All statistical analyses were implemented in R environment [26], using ARTool R package [27,28]. The package factoextra was used for the principal component analysis (PCA). Graph and plot reports were created with the ggplot2 package [29].

## Figures and Tables

**Table 1:** Base parameters of Aglianico, Barbera, Gaglioppo, and Nerello wines before the treatments.

Total Acidity (mg/L)	Aglianico			Barbera			Gaglioppo			Magliocco			Nerello		
EtOH (%)	15.11	±	0.14	13.34	±	0.06	13.05	±	0.01	10.68	±	0.02	11.47	±	0.02
pH	3.25	±	0.02	3.77	±	0.01	3.55	±	0.01	3.84	±	0.08	3.77	±	0.01
free SO_2_ (mg/L)	12.65	±	0.44	19.77	±	0.80	11.1	±	0.60	7.15	±	0.05	2.40	±	0.01
total SO_2_ (mg/L)	47.05	±	0.66	37.45	±	0.10	61.60	±	0.46	26.4	±	0.16	16.75	±	1.90
total acidity (mg/L)	7.17	±	0.04	4.59	±	0.05	5.57	±	0.10	4.65	±	0.01	4.20	±	0.05
volatile acidity (mg/L)	0.51	±	0.02	0.69	±	0.02	0.93	±	0.02	0.61	±	0.01	0.64	±	0.01
sugar (mg/L)	1.95	±	0.12	2.3	±	0.11	1.92	±	0.09	1.39	±	0.01	1.7	±	0.14
monomeric anthocyanins (mg/L)/BSA Reactive Tannins (mg/L)	0.31			2.40			0.03			0.22			0.05		

**Table 2:** Main CIEL*a*b* values of wines in response to oxidative tests *.

Name	L*	a(u)*	b(v)*	Chromacity	Hue Angle	∆E
Aglianico						
control	41.5 ± 0.31	40.15 ± 0.1	9.325 ± 0.05	41.25 ± 0.01	41.25 ± 0.02	
sat	37.67 ± 0.05	42.57 ± 0.05	11.72 ± 0.05	44.17 ± 0.05	15.42 ± 0.05	5.13 ± 0.19
H_2_O_2_	36.42 ± 0.09	43.25 ± 0.05	11.37 ± 0.05	44.75 ± 0.05	14.75 ± 0.05	6.29 ± 0.18
AtCH	36.57 ± 0.20	43.75 ± 0.70	11.15 ± 0.17	45.12 ± 0.72	14.3 ± 0.00	6.41 ± 0.14
Barbera						
control	51.8 ± 0.12	28.775 ± 0.05	10.7 ± 0.08	30.7 ± 0.08	20.4 ± 0.08	
sat	40.9 ± 0.2	41.025 ± 0.12	12.475 ± 0.05	42.87 ± 0.09	16.95 ± 0.1	16.49 ± 0.18
H_2_O_2_	39.52 ± 0.09	41.72 ± 0.05	11.22 ± 0.05	43.17 ± 0.09	15.05 ± 0.05	17.85 ± 0.09
AtCH	39.67 ± 0.20	42.8 ± 0.11	11.52 ± 0.09	44.35 ± 0.05	15.07 ± 0.15	18.56 ± 0.11
Gaglioppo						
control	71.25 ± 0.45	9.3 ± 0.10	5.63 ± 0.20	10.86 ± 0.05	31.12 ± 1.19	
sat	70.27 ± 0.05	11.17 ± 0.05	9.67 ± 0.05	14.77 ± 0.05	40.77 ± 0.05	4.59 ± 0.16
H_2_O_2_	70.37 ± 0.05	11.0 ± 0.8	9.3 ± 0.00	14.4 ± 0.8	40.2 ± 0.14	4.18 ± 0.13
AtCH	69.85 ± 0.75	11.27 ± 0.05	9.52 ± 0.32	14.8 ± 0.18	40.17 ± 1.01	4.63 ± 0.12
Magliocco						
control	71.42 ± 0.12	9.35 ± 0.05	8.52 ± 0.09	12.65 ± 0.19	42.3 ± 0.21	
sat	67.45 ± 0.05	14.5 ± 0.00	6.05 ± 0.1	15.67 ± 0.05	22.725 ± 0.29	6.96 ± 0.13
H_2_O_2_	69.22 ± 0.32	12.42 ± 0.32	6.65 ± 0.05	14.1 ± 0.29	28.17 ± 0.74	4.24 ± 0.43
AtCH	65.27 ± 0.98	15.92 ± 0.26	5.1 ± 0.32	16.72 ± 0.36	17.65 ± 0.83	9.68 ± 0.78
Nerello						
control	75.35 ± 0.7	4.2 ± 0.27	4.975 ± 0.05	6.575 ± 0.15	50.075 ± 2.11	
sat	74.15 ± 0.13	6.5 ± 0.00	5.7 ± 0.00	8.62 ± 0.05	41.37 ± 0.09	2.83 ± 0.8
H_2_O_2_	74.5 ± 0.18	5.92 ± 0.05	5.65 ± 0.12	8.17 ± 0.09	43.65 ± 0.23	2.16 ± 0.46
AtCH	73.8 ± 0.5	6.27 ± 0.09	5.55 ± 0.17	8.37 ± 0.20	41.62 ± 0.55	2.82 ± 0.80

* All treated wines differed significantly from the control (*p* < 0.005).

## Data Availability

Not applicable.

## Appendix A

**Table A1:** Monomeric anthocyanins content (mg/L) of wines before and after oxidative treatments.

	**Aglianico**											
	Control			AtCH			H_2_O_2_			sat		
Dp3glc	33.29	±	0.53	13.33	±	0.49	17.08	±	0.61	17.28	±	0.37
Cy3glc	1.12	±	0.02	0.47	±	0.06	0.64	±	0.08	0.58	±	0.13
Pt3glc	40.07	±	1.34	6.79	±	0.19	8.39	±	0.35	8.47	±	0.51
Pn3glc	10.29	±	0.47	3.58	±	0.67	5.41	±	0.19	5.85	±	0.06
Mv3glc	370.44	±	2.50	188.06	±	3.02	222.50	±	16.39	229.24	±	8.24
Mv3acglc	11.75	±	0.60	2.31	±	0.17	6.11	±	1.15	2.60	±	0.23
Mv3cmglc	23.77	±	1.72	4.46	±	1.73	5.48	±	0.67	5.17	±	0.61
Tot mon anth	490.73	±	3.08	219.00	±	5.19	265.62	±	19.15	269.19	±	8.44
	**Barbera**											
	Control			AtCH			H_2_O_2_			sat		
Dp3glc	87.24	±	0.90	64.71	±	1.72	63.15	±	0.87	71.24	±	1.85
Cy3glc	6.95	±	0.92	4.38	±	0.92	2.19	±	0.24	3.64	±	2.04
Pt3glc	119.70	±	1.09	89.83	±	0.94	87.03	±	1.21	98.57	±	1.54
Pn3glc	42.09	±	0.52	31.21	±	2.53	32.80	±	0.09	35.23	±	1.40
Mv3glc	883.90	±	1.97	692.66	±	6.52	682.79	±	9.52	762.20	±	0.52
Mv3acglc	139.47	±	3.07	101.87	±	2.92	108.71	±	0.75	112.60	±	3.72
Mv3cmglc	75.47	±	0.65	57.04	±	1.77	50.94	±	2.76	55.10	±	2.63
tot mon anth	1354.81	±	6.78	1041.70	±	8.88	1027.62	±	9.45	1138.58	±	9.59
	**Gaglioppo**											
	Control			AtCH			H_2_O_2_			sat		
Dp3glc	1.63	±	0.05	0.87	±	0.04	1.06	±	0.09	1.59	±	0.04
Cy3glc	0.97	±	0.03	0.35	±	0.05	0.46	±	0.15	0.92	±	0.06
Pt3glc	2.66	±	0.18	1.63	±	0.06	1.82	±	0.27	2.29	±	0.13
Pn3glc	2.53	±	0.15	1.09	±	0.10	1.42	±	0.03	2.33	±	0.24
Mv3glc	21.76	±	0.67	11.99	±	0.12	14.99	±	0.31	18.10	±	0.46
Mv3acglc	1.62	±	1.50	0.39	±	0.12	0.39	±	0.05	0.51	±	0.12
Mv3cmglc	5.32	±	0.80	1.70	±	0.30	2.42	±	0.86	3.96	±	1.23
tot mon anth	36.48	±	2.07	18.03	±	0.33	22.57	±	1.12	30.07	±	0.95
	**Magliocco**											
	Control			AtCH			H_2_O_2_			sat		
Dp3glc	6.93	±	0.18	3.21	±	0.17	1.52	±	0.14	5.63	±	0.23
Cy3glc	2.26	±	0.28	1.11	±	0.05	0.50	±	0.03	1.83	±	0.17
Pt3glc	10.96	±	0.07	5.13	±	0.23	2.73	±	0.05	9.13	±	0.08
Pn3glc	9.72	±	0.72	4.50	±	0.15	2.42	±	0.13	8.67	±	0.10
Mv3glc	167.91	±	0.57	86.66	±	1.09	53.12	±	0.50	146.79	±	0.81
Mv3acglc	7.56	±	0.62	3.56	±	0.25	2.04	±	0.59	6.98	±	0.56
Mv3cmglc	17.72	±	1.39	7.84	±	0.32	5.16	±	0.70	14.45	±	1.98
tot mon anth	223.06	±	1.25	112.00	±	1.44	67.50	±	0.19	193.48	±	3.47
	**Nerello**											
	Control			AtCH			H_2_O_2_			sat		
Dp3glc	4.48	±	0.14	3.92	±	0.02	4.03	±	0.11	3.64	±	0.13
Cy3glc	2.74	±	0.08	2.44	±	0.11	2.55	±	0.02	2.94	±	0.10
Pt3glc	4.60	±	0.17	4.07	±	0.04	4.43	±	0.06	3.07	±	0.10
Pn3glc	5.26	±	0.21	4.44	±	0.17	5.04	±	0.05	3.90	±	0.21
Mv3glc	20.03	±	1.09	18.97	±	0.42	21.40	±	0.12	18.46	±	1.21
Mv3acglc	0.13	±	0.02	0.18	±	0.06	0.17	±	0.02	0.29	±	0.01
Mv3cmglc	0.31	±	0.10	0.13	±	0.06	0.26	±	0.25	0.22	±	0.02
tot mon anth	37.55	±	1.40	34.15	±	0.36	37.88	±	0.18	32.70	±	0.89

Dp3glc = delphinidin 3-glucoside, Cy3glc = cyanidin 3-monoglucoside, Pt3glc = petunidin 3-monoglucoside, Pn3glc = peonidin 3-monoglucoside, Mv3glc = malvidin 3-glucoside, Mv3acglc = malvidin 3-(6^II^-acetyl)-glucoside, Mv3cmglc = malvidin 3-(6^II^-coumaroyl)-glucoside, tot mon anth = total amount of monomeric anthocyanins.

## References

[1] Du Toit W.J., Marais J., Pretorius I.S., Du Toit M. (2006). Oxygen in must and wine: A review. S. Afr. J. Enol. Vitic..

[2] Waterhouse A.L., Laurie V.F. (2006). Oxidation of wine phenolics: A critical evaluation and hypotheses. Am. J. Enol. Vitic..

[3] Waterhouse A.L., Sacks G.L., Jeffery D.W. (2016). Understanding Wine Chemistry.

[4] Picariello L., Gambuti A., Picariello B., Moio L. (2017). Evolution of pigments, tannins and acetaldehyde during forced oxidation of red wine: Effect of tannins addition. LWT.

[5] Deshaies S., Cazals G., Enjalbal C., Constantin T., Garcia F., Mouls L., Saucier C. (2020). Red Wine Oxidation: Accelerated Ageing Tests, Possible Reaction Mechanisms and Application to Syrah Red Wines. Antioxidants.

[6] Sheridan M.K., Elias R.J. (2015). Exogenous acetaldehyde as a tool for modulating wine color and astringency during fermentation. Food Chem..

[7] Teng B., Hayasaka Y., Smith P.A., Bindon K.A. (2019). Effect of Grape Seed and Skin Tannin Molecular Mass and Composition on the Rate of Reaction with Anthocyanin and Subsequent Formation of Polymeric Pigments in the Presence of Acetaldehyde. J. Agric. Food Chem..

[8] Gambuti A., Picariello L., Rinaldi A., Moio L. (2018). Evolution of Sangiovese wines with varied tannin and anthocyanin ratios during oxidative aging. Front. Chem..

[9] Ferreira V., Carrascon V., Bueno M., Ugliano M., Fernandez-Zurbano P. (2015). Oxygen consumption by red wines. Part I: Consumption rates, relationship with chemical composition, and role of SO_2_. J. Agric. Food Chem..

[10] Jenkins T.W., Howe P.A., Sacks G.L., Waterhouse A.L. (2020). Determination of Molecular and “Truly” Free Sulfur Dioxide in Wine: A Comparison of Headspace and Conventional Methods. Am. J. Enol. Vitic..

[11] Harbertson J.F., Picciotto E.A., Adams D.O. (2003). Measurement of polymeric pigments in grape berry extract sand wines using a protein precipitation assay combined with bisulfite bleaching. Am. J. Enol. Vitic..

[12] Waterhouse A.L., Zhu J. (2020). A quarter century of wine pigment discovery. J. Sci. Food Agric..

[13] Pérez-Magariño S., González-San José M.L. (2004). Evolution of flavanols, anthocyanins, and their derivatives during the aging of red wines elaborated from grapes harvested at different stages of ripening. J. Agric. Food Chem..

[14] Avizcuri J.M., Sáenz-Navajas M.P., Echávarri J.F., Ferreira V., Fernández-Zurbano P. (2016). Evaluation of the impact of initial red wine composition on changes in color and anthocyanin content during bottle storage. Food Chem..

[15] Howe P.A., Worobo R., Sacks G.L. (2018). Conventional measurements of sulfur dioxide (SO_2_) in red wine overestimate SO_2_ antimicrobial activity. Am. J. Enol. Vitic..

[16] Mokrzycki W.S., Tatol M. (2011). Colour difference∆ E-A survey. Mach. Graph. Vis..

[17] Sacks G.L., Howe P.A., Standing M., Danilewicz J.C. (2020). Free, Bound, and Total Sulfur Dioxide (SO_2_) during Oxidation of Wines. Am. J. Enol. Vitic..

[18] Vrhovsek U., Mattivi F., Waterhouse A.L. (2001). Analysis of red wine phenolics: Comparison of HPLC and spectrophotometric methods. Vitis.

[19] Timberlake C.F., Bridle P. (1976). Interactions between anthocyanins, phenolic compounds, and acetaldehyde and their significance in red wines. Am. J. Enol. Vitic..

[20] Poncet-Legrand C., Cabane B., Bautista-Ortín A.B., Carrillo S., Fulcrand H., Pérez J., Vernhet A. (2010). Tannin oxidation: Intra-versus intermolecular reactions. Biomacromolecules.

[21] Forino M., Picariello L., Lopatriello A., Moio L., Gambuti A. (2020). New insights into the chemical bases of wine color evolution and stability: The key role of acetaldehyde. Eur. Food Res. Technol..

[22] Rivas-Gonzalo J.C., Bravo-Haro S., Santos-Buelga C. (1995). Detection of compounds formed through the reaction of malvidin 3-monoglucoside and catechin in the presence of acetaldehyde. J. Agric. Food Chem..

[23] Zhang X.K., Lan Y.B., Huang Y., Zhao X., Duan C.Q. (2020). Targeted metabolomics of anthocyanin derivatives during prolonged wine aging: Evolution, color contribution and aging prediction. Food Chem..

[24] Escribano-Bailón T., Álvarez-García M., Rivas-Gonzalo J.C., Heredia F.J., Santos-Buelga C. (2001). Color and stability of pigments derived from the acetaldehyde-mediated condensation between malvidin 3-O-glucoside and (+)-catechin. J. Agric. Food Chem..

[25] Organisation Internationale de la Vigne et du Vin Compendium of International Methods of wine And Must Analysis. https://www.oiv.int.

[26] R Core Team (2020). R: A Language and Environment for Statistical Computing.

[27] Kay M., Wobbrock J. (2020). ARTool: Aligned Rank Transform for Nonparametric Factorial ANOVAs, R Package Version 0.10.8. https://github.com/mjskay/ARTool.

[28] Wobbrock J., Findlater L., Gergle D., Higgins J. The Aligned Rank Transform for Nonparametric Factorial Analyses Using Only ANOVA Procedures. Proceedings of the ACM Conference on Human Factors in Computing Systems (CHI ‘11).

[29] Kassambara A. (2017). Practical Guide to Principal Component Methods in R: PCA, M (CA), FAMD, MFA, HCPC, Factoextra.

